# Exploring the Use of a Digital Platform for Cancer Patients to Report Their Demographics, Disease and Therapy Characteristics, Age, and Educational Disparities: An Early-Stage Feasibility Study

**DOI:** 10.3390/curroncol30080551

**Published:** 2023-08-12

**Authors:** Dimitra Galiti, Helena Linardou, Sofia Agelaki, Athanasios Karampeazis, Nikolaos Tsoukalas, Amanda Psyrri, Michalis Karamouzis, Konstantinos N. Syrigos, Alexandros Ardavanis, Ilias Athanasiadis, Eleni Arvanitou, Stavroula Sgourou, Anastasia Mala, Christos Vallilas, Ioannis Boukovinas

**Affiliations:** 1 Clinic of Oral Diagnosis and Radiology, School of Dentistry, National and Kapodistrian University of Athens, 15772 Athens, Greece; 2 Hellenic Society of Medical Oncology, 11475 Athens, Greece

**Keywords:** cancer, digital health, patients’ characteristics, age, educational disparities, socioeconomic issues

## Abstract

Introduction: The increasing burden of cancer, the development of novel therapies, and the COVID-19 pandemic have made cancer care more complex. Digital innovation was then pushed toward developing platforms to facilitate access to cancer care. Age, education, and other disparities were, however, shown to limit the use of the digital health innovation. The aim of this early-stage feasibility study was to assess whether Greek cancer patients would register at CureCancer and self-report their demographics, disease and therapy characteristics, and socioeconomic issues. The study was organized by the Hellenic Society of Medical Oncology. Methods: Patients from nine cancer centers were invited to register on the CureCancer platform and complete an anonymous questionnaire on demographics, disease and therapy characteristics, and socioeconomic issues. Patients were also encouraged to upload, in a secure area for them, their medical files and share them with their physicians. They were then asked to comment on their experience of registration and how easy it was to upload their medical files. Results: Of the 159 patients enrolled, 144 (90.56%) registered, and 114 of those (79.16%) completed the questionnaire, suggesting that the study is feasible. Users’ median age was 54.5 years, and 86.8% of them were university and high school graduates. Most patients (79.8%) reported their specific type of cancer diagnosis, and all reported their therapy characteristics. Breast and lung cancers were the most common. A total of 87 patients (76.3%) reported being on active cancer therapy, 46 (40.4%) had metastatic disease, and 51 (44.7%) received supportive care medications. Eighty-one (71.05%) patients received prior cancer therapies, and twenty-seven recalled prior supportive care medications. All patients reported visiting non-oncology Health Care Professionals during the study. Nineteen of 72 (26.39%) patients who worked prior to cancer diagnosis changed work status; 49 (42.98) patients had children under 24 years; and 16 (14%) patients lived alone. Nine (7.9%) patients were members of patient associations. Registration was “much/very much” easy for 98 (86.0%) patients, while 67 (58.8%) had difficulties uploading their files. Patients commented on the well-organized data access, improved communication, feeling safe, medication adherence, interventions from a distance, and saving time and money. Over 80% of patients “preferred the digital way”. Discussion: A total of 114 patients succeeded in registering on the digital platform and reporting their demographics, disease and therapy characteristics, and socioeconomic issues. Age and educational disparities were disclosed and highlighted the need for educational programs to help older people and people of lower education use digital innovation. Health care policy measures would support patients’ financial burden associated with work changes, living alone, and children under 24 years old at school or college. Policy actions would motivate patients to increase their participation in patient associations. According to the evidence DEFINED framework, the number of patients, and the focus on enrollment, engagement, and user experience, the study fulfills actionability level criterion 1.

## 1. Introduction

Globally, 43.8 million people live with cancer. A 60% increase in cancer cases is estimated by 2040, with 29.4 million patients per year requiring therapy and a demand for increased costs [1,2]. In Canada, one in two men or women is anticipated to develop cancer during their lifetime [3], and in the UK, one person is diagnosed with cancer every two minutes [4]. The increasing burden of new cancer cases and cancer survivors and the development of novel therapies have made cancer clinical practice and supportive care more complex and expensive. Difficulties in having access to patients’ medical histories, communication between patients, oncologists, and other Health Care Professionals (HCPs), travel restrictions related to the global pandemics, the unemployment risk of all employed working-age cancer survivors, and financial or social/family problems can further limit the ability to provide early and effective cancer care. Early initiation of supportive care has been shown to reduce cancer costs by up to 33%, while embedding the patient perspective is considered a hallmark of quality clinical care and research [2,5,6].

Digital innovation was pushed toward the development of patient portals and platforms, particularly after the advent of the COVID-19 pandemic, to overcome the difficulties and improve clinical practice [7,8,9,10]. The digital innovation is particularly useful when patients live outside the cancer center and traveling is not easy due to individuals’ being unwell, work, or family/children care, and other problems [7,8].

A digital health care solution for effective supportive cancer care should have: (1) a well-informed patient, having direct access to his medical files and sharing them with the cancer care team; (2) an accurate symptoms’ record, in real-world and real-time, to share with HCPs and facilitate early management; (3) the potential to provide live communication between patients and HCPs and facilitate patient assessment, monitoring, and management with telemedicine; and (4) the availability of the accurate patient files to complement clinical trials, assist treatment decision-making, and to advise the health care policy and economic modelers [11].

Compliance with data privacy standards is also necessary. Furthermore, digital health equity is a critical domain; language support, digital literacy, cultural appropriateness, and technical accessibility should be considered [12]. Disparities in age, digital health literacy, race, income, rural geography, ethnicity, education, access to patients’ data during telehealth visits, and communication-related disabilities have been shown to limit the use of digital innovation [13,14,15]. The characterization of those disparities can help to define the interventions that may be needed to increase the use of health technology and close the digital divide in each country.

Challenges for the evaluation of digital health solutions exist. A huge challenge for end users, patients, and providers, such as healthcare professionals and hospital administrators, is how to determine a new solution’s credibility. In the early stages of development innovators seek to establish product usability, feasibility, and efficacy. Surveys and/or interviews are often employed, which are low-cost, efficient tools to collect attitudes, user experience, and suitability. Controversy exists regarding the appropriate number of participants [12].

A rigorous and rapid evidence assessment in digital health with the evidence DEFINED framework was recently elaborated to facilitate standardized, rapid, and rigorous Digital Health Innovation (DHI) evidence assessment and guide digital health solution providers who wish to generate evidence that drives DHI adoption [16].

The present early-stage feasibility study aimed to assess whether Greek cancer patients would register at CureCancer and self-report their demographics, disease and therapy characteristics, and socioeconomic issues. The assessment of basic patients’ sociodemographic and clinical characteristics has been reported as fundamental to quickly outlining efficient, supportive consideration and patient management [17]. User experience was also collected. The study was organized by the Hellenic Society of Medical Oncology-HeSMO.

About CureCancer, www.curecancer.eu, accessed on 10 August 2023. CureCancer is a patient-driven digital health platform that can enable patients: (1) to self-create their medical profile and report their sociodemographic, disease, and therapy characteristics; (2) to record their symptoms in real-world and real-time and share files and symptoms with their physicians; and (3) to communicate with physicians with a video call to receive visual care. CureCancer was inspired by Dr Dimitra Galiti, a senior PhD dental student who experienced difficulties in communication with physicians and accessing patients’ medical histories during her dental oncology clinical practice. CureCancer is registered at Elevate Greece, which is the national startup point of the Greek startup ecosystem, https://elevategreece.gov.gr/, accessed on 10 August 2023.

CureCancer was selected to participate in the program SymbIASIS “connecting startups with hospitals”. This program is under the European Institute of Innovation and Technology, EIT Health, and is supervised in Greece by the National Documentation Center, EKT, with the collaboration of the Archimedes Center for Innovation and Entrepreneurship of Greece, National and Kapodistrian University of Athens, https://www.ekt.gr/en/news/27383, accessed on 10 August 2023. Today, CureCancer is connected with the Oncology Clinic of ATTIKON Hospital, the largest hospital in Athens, and SOTIRIA Hospital, another large Athens Hospital. Within 3 months of collaboration, we have collected 1023 patients’ questionnaires. The Dental Oncology Clinic of the General Hospital of LARISA, a smaller city hospital, has just joined, and two more dental oncology clinics have asked to join, one from a hospital in Athens and one from a hospital on mainland Greece. On 23 May 2023, CureCancer presented its work at the session “Success Stories and a how-to guide” organized by the EKT and EIT Health, https://www.ekt.gr/en/events/29032, accessed on 10 August 2023.

## 2. Patients and Methods

### 2.1. Patients

Eligibility criteria: Patients who expressed an interest in participating in the study were included if they were: (1) 18 years of age or older; (2) diagnosed with cancer at least one month earlier, when they were well-embarked on a treatment plan; (3) were on active cancer treatment and thereafter; (4) had over one year of prognosis; (5) self-reported internet knowledge; (6) could understand the Greek language to read and understand the forms and questionnaire; and (7) agreed to sign the information and consent forms. Patients were excluded if they lacked the capacity to give informed consent due to psychopathology, cognitive dysfunction, learning difficulties, or other problems.

Ethical approval: The study protocol, including an anonymous questionnaire on sociodemographic, disease, and therapy characteristics to be completed by patients, was approved by the Committee of Research and Ethics of all participating cancer centers.

### 2.2. Methods

The Hellenic Society of Medical Oncology-HeSMO, www.hesmo.gr, accessed on 10 August 2023, asked their members to participate in the study. Nine cancer centers responded. Eligible patients were invited by their oncologists to participate in the study and sign the information letter and the consent form. An electronic consent form was also available online. Patients’ invitations depended on the availability of the oncologists, as study-dedicated personnel were not hired due to a lack of financial support. Patients were given written instructions on how to access the platform. The benefits of the use of digital innovation, as explained to patients, were the well-organized medical files and data, available from anywhere and anytime, to facilitate their communication with physicians and receive early and effective care at home, save time and travel costs, and reduce the risk of infection.

The patients who signed the consent form were asked to register on the platform and complete an anonymous questionnaire at home at their convenience. They were free to collaborate with the family members while completing the questionnaire. Their experience using the platform was assessed with appropriate questions.

Patients were encouraged to upload their medical files in a secure area, for example, blood tests, radiographs, or clinical photos, and share their files with their health care professionals.

Following the completion of the study, the results of the anonymous questionnaires were sent from CureCancer to HeSMO for evaluation.

Questionnaire: The questionnaire was developed and agreed upon by all authors to help patients self-report and record their demographics, disease characteristics, and therapies. It consisted of two parts. In the 1st part, patients were asked to complete questions on demographic, disease and cancer therapy characteristics, and socioeconomic issues. In the 2nd part, patients were asked to answer questions as to whether their registration on the platform was easy. Furthermore, patients who had uploaded their files on the platform and communicated with their physicians in a secure area were asked to report on their experience with this feature. The exact number of patients who used the platform to upload files and communicate with physicians is not known since this function takes place in a secure environment.

Patients were also asked to suggest any changes to improve the platform. Physicians, too, were asked to comment on their communication with patients and other colleagues, whether they feel that the digital platform can improve their clinical practice, and if they would prefer the digital way. Oncologists were also asked to suggest improvements to the platform.

### 2.3. Endpoints

Cancer patients’ demographic, disease, and therapy characteristics, as well as socioeconomic problems following cancer diagnosis, were explored.

The digital innovation would be accepted as feasible if >70% of the registered patients would complete the questionnaire. The evidence DEFINED framework actionability level criteria were used to assess the actionability level of the platform [16].

### 2.4. Data Analysis

Patients’ demographics, disease characteristics, therapies, socioeconomic profile, and questions on the registration and use of the platform were primarily analyzed using descriptive statistics. For categorical variables, absolute and relative frequencies were provided, while continuous variables were described by N, mean, and standard deviation, or median and range. Any possible associations were investigated using the chi-square and *t* tests. Statistical Analysis was conducted in Stata 15.1 [18].

The STROBE checklist was followed throughout the writing of this manuscript [19].

## 3. Results

From January to December 2020, 159 patients signed the consent form to participate, and 144 of those (90.56%) registered on the platform. Of all 144 patients, 114 (79.16%) completed the questionnaire (Table 1).

### 3.1. Patients and Disease Characteristics

The median age was 54.5 years, while most patients (86.8%) were university and high school graduates. Breast and lung cancers were the most common. Twenty-three patients (20.2%) did not report their cancer type, and five patients (4.4%) reported having multiple cancers, not otherwise specified. Forty-six patients (40.4%) had metastatic disease.

### 3.2. Cancer Therapy Characteristics

Eighty-seven (76.3%) patients were on active cancer therapy, with 48 of those patients (42.1%) receiving chemotherapy. Twenty-seven patients (23.7%) did not receive therapy during the study period.

### 3.3. Supportive Care

Fifty-one patients (44.7%) reported receiving supportive care, such as antiemetics, pain medications, anti-depressants, bone targeting agents, and a complementary medical diet.

### 3.4. Visiting Non-Oncology HCPs

During the study period, all patients visited at least one non-oncology HCP, while 47 patients (41.2%) visited 2–9 different non-oncology HCPs. The total number of HCPs from different specialties was 208 (mean number per patient = 1.82). The mean number of HCPs was higher, though not significantly, among patients under active treatment compared to no current therapy group (1.92 vs. 1.51; *p* = 0.18, *t*-test) (Table 1). The total number of visits by each patient to each HCP was not recorded.

### 3.5. Prior Cancer Therapies

Eighty-one patients recalled having received prior cancer therapies, such as surgery (*n* = 62), chemotherapy (*n* = 44), radiotherapy (*n* = 5), hormonal therapy (*n* = 5), and targeted therapy (*n* = 1). Fifty patients had received more than one type of therapy. Furthermore, 27 patients recalled that they had also received supportive care medications, including anti-depressants, pain medications, a medical diet, and medications for bone metastases.

### 3.6. Working Status

Nineteen (26.39%) of 72 patients who worked prior to cancer diagnosis changed their work status following cancer. Seven patients reported that their contracts were not renewed after cancer diagnosis; two patients changed work because of cancer therapy; and six patients retired after cancer diagnosis.

### 3.7. Other Social Profile Characteristics

Sixteen patients (14%) lived alone; forty-nine (43%) had children younger than 24 years of age; and nine (7.9%) were members of patients’ associations.

### 3.8. Registration and Use of the Platform

A total of 98 of the 114 patients (86.0%) reported their registration at the platform as “very to very much” easy. File uploading was reported as “very to very much easy” by 47 (41.2%) patients. Ninety (78.9%) patients preferred the digital way, and 99 (86.8%) will introduce it to others (Table 2).

### 3.9. Patients’ Comments

Fifty-four patients commented on the “easy access, well-organized data, feeling safe, reduced visits, saving time and money, no need to count the days/time for my medication, minimized the risk of COVID-19 infection, uploaded photos to inform the doctor, and receive a direct, fast, and effective response”. Twelve patients asked for “a user-friendlier environment”, six had “nothing to change”, two asked “to receive newsletters”, one requested for “a mobile application”, and another “complained of the many questions”. Sixty patients did not make any comments.

At this point, may we present an example of patient care through the platform. A husband called the oral medicine doctor in Athens to ask for help and said to the doctor “I am losing my wife”. The patient’s wife had just completed chemoradiotherapy in Athens and had returned to her hometown. She was vomiting all day and would not take any food in her mouth. The husband uploaded a photo of his wife’s mouth and gave access to the doctor to check if the problem was in the mouth. The oral mucosa was intact, and the patient was advised to call the radiation oncologist, who, in turn, advised her to quit the pain medication. The patient’s nausea and vomiting were related to the pain medication’s toxicity. Two days later, the woman completely recovered and had three regular meals.

### 3.10. Physicians’ Comments

Physicians commented that the use of the platform improved their clinical practice and their communication with patients and other physicians. Reduced risk of infection, hospital visits, paperwork, and clinical burden; fast, easy, and safe access to well-organized data; direct communication anytime, from anywhere; early reporting of symptoms; and timely management were other comments.

A more user-friendly digital environment/access to the platform, the potential/possibility to collect their patients’ data in aggregates, and receiving a message when a patient uploaded a new file, symptom, or alert sign were requested.

The platform was accepted as feasible since >70% of the registered patients completed the questionnaire. According to the evidence-based DEFINED framework and the focus on enrollment, engagement, and user experience of 114 patients, the CureCancer platform was assigned the actionability level criterion one [16].

## 4. Discussion

The use of the digital platform.

Over 70% of the registered patients completed the questionnaire and disclosed important demographics and other cancer-related characteristics. The small number of patients and the focus of the study on enrollment, engagement, and user experience fulfilled actionability level criterion one [16].

### 4.1. CureCancer Users Were Younger Patients with Higher Education

The median age of our patient users was about a decade younger than the median global cancer age [1]. In the present patient group, older age seemed to be a barrier, which may have limited the use of the digital platform.

Younger patients were significantly more likely to be able to assess information online, use inpatient portals, and have an interest in communicating with their health care team remotely, as reported in other studies [13,20,21]. Walker et al. [13] showed that patients aged 18–29 used an inpatient portal more often than patients over 60 years old. The authors recognized that an educational intervention may be important to help older users overcome age disparities. Aljabri et al. [20] reported that portal adoption was also associated with patients who were young, among other determinants such as higher income, female gender, higher disease severity, and other issues. In a study that was performed using telephone interviews, older adults reported digital access and digital literacy as barriers to engaging in telemedicine [21].

Furthermore, younger patients or those with a higher education level at an urban cancer center were significantly more likely to own a smartphone, access health information online, have an interest in communicating with their health care team remotely, and know how to upload an app compared with older patients and those with a lower level of education [14]. That center reported offering classes to teach patients and caregivers how to sign up for and access their electronic health records. A telehealth task force was created to walk patients through the process of conducting a telehealth visit.

In the present study, most of our patients were of higher education: university and high school graduates. Similarly, all participants were college graduates in another study, while the median age of patients was 55 years, as in our study [22]. Younger patients were the ones who expressed an interest and self-reported their internet literacy and were “selected” to be included in the study, as in other research projects [10,23].

Performing structured interviews regarding the barriers and facilitators of patients’ current and desired use of mHealth technology for health services, Potdar et al. [24] reported that educational achievement and a college-level degree were significant predictors of acceptability and use of digital health innovations.

### 4.2. Educational Interventions to Overcome the Gaps of Age and Internet Literacy

An 18-point “digital universal precautions” mandate for health care organizations committed to addressing and facilitating eHealth literacy has recently been proposed [25]. Two of the eighteen precautions focused on ease of use and making health literacy a standard, encouraging the development of educational material and tools. Multifaceted education and training of the staff, as well as handouts and website content to optimize patients’ engagement, were initiated by others. Both staff and patients expressed positive feedback on the use of telemedicine and its benefits [26].

Developing strategies to improve patient knowledge and skills when using telehealth services is likely to be a new and essential function in oncology care teams to optimize the use of telemedicine. The authors have agreed that a comprehensive assessment of patient-level barriers, including readiness to use telehealth, disabilities that limit telehealth use, and limited literacy, must be understood and mitigated. Telehealth access can be improved through interventions such as patient-level training, simple designs, and engaging informal caregivers. Education and training for digital and telehealth tools must be built into clinical workflows to address disparities in access. This training is often time-consuming and resource-intensive, but it is an investment in excellent cancer care that can increase patient engagement [13,14,23,24,25,26,27].

### 4.3. Most of Our Patients Were Well Informed about Their Cancer Type

Most of our patients/users (79.8%) were well-informed and reported the specific type of their cancer diagnosis. Breast and lung cancers were the most common cancer types, as anticipated, while the lack of prostate cancer reporting was interesting and could be related to the younger age of our cohort [1]. Well-informed patients and having direct access to their medical files, which they can share with caring physicians, are important components of routine cancer clinical practice to facilitate early successful management [5,6,8,11,26,27]. In a recent study, patients who knew the characteristics of their illness and treatments more frequently used adaptive coping strategies [17].

The anonymous nature of the questionnaires, which patients completed at their convenience, did not allow us to check the reliability of the cancer types given by patients. It is our experience, however, during our daily clinical practice, that most patients and/or the accompanying family members or caregivers know the accurate cancer type. Furthermore, during our ongoing study in the SymbIASIS program, patients know their cancer type, as the study nurse checks and confirms for each patient. Today, by 12 July 2023, we have collected 1023 patient-completed questionnaires, and all cancer-type diagnoses given by the patients were correct.

Five patients reported having different cancers without identifying the cancer types or whether those cancers represented metastatic disease from one primary cancer. This information cannot be further documented. Twenty-three patients did not record their cancer-type diagnosis. The reason for that could be a lack of knowledge by those patients or a lack of help from a family member. Indeed, 16 patients lived alone, with no family at home to help. Another reason for not completing the question on the cancer type might be that some patients skipped that question by mistake since the questions on that questionnaire were not mandatory.

### 4.4. Patients’ Knowledge about Their Therapy Characteristics

All patients completed the information on their treatments. The level of accuracy of this information cannot be documented since there was no dedicated nurse to check the answers and all questionnaires were anonymous.

### 4.5. Patients Highlighted the Role and the Important Need to Receive Supportive Care by Non-Oncology HCPs

Fifty-one patients needed supportive care medications, and all of them visited at least one non-oncology health care professional, highlighting the role of supportive care in cancer and the importance of collaboration with the non-oncology HCPs. This finding highlights another role for digital health, which is to facilitate collaboration between oncologists and non-oncology HCPs, which can minimize the risk of inappropriate polypharmacy [28,29].

### 4.6. Patients Highlighted the Benefits of Digital Health in Their Own Words

Sense of safety, treatment adherence, reduced hospital visits and infection risk, saving time and money, and good communication with their doctors, which were reported by the patients, are aligned with the benefits of the digital innovations in cancer care, including medication adherence, particularly when using oral antineoplastics, as commented on or reported by other authors [8,11,15,23,30,31].

### 4.7. Patients Disclosed Socioeconomic Challenges that Affected the Family, Too

A cancer diagnosis was related to changes at work, such as retirement, no contract renewal, or job change. In other studies, cancer diagnosis and therapy have also been associated with negative consequences for employment, financial problems, increased financial toxicity, and social well-being [32,33,34]. Living alone patients, who may need a paid caregiver, as well as children at school and university, who may need financial support for their education costs, can also add to patients’ and families burdens and financial toxicities. In Greece, families may continue to support students during their university studies.

Although patient communities and non-profit associations have been reported as the main environments to connect with others and obtain trusted information [23], few patients in the present study participated in patient communities. Cultural reasons and/or lack of referral mechanisms and adequate channels of communication between patients and non-profit community-based providers and patients’ associations may be some of the reasons for the above difference [35].

### 4.8. Physicians Wish to Continue Using CureCancer

Physicians commented on the improved communication with patients and colleagues, enhancing their clinical practice, and preferred to maintain the use of digital health innovation after the pandemic, as has been reported in other studies [36,37,38]. The authors of those studies agreed that virtual care will have a continuing role in providing access that is more convenient, while telehealth is well accepted by various cancer care clinicians. The importance of overcoming the digital divide through the development of patient educational materials and ensuring that policies are supportive of oncology telehealth was highlighted. Reimbursement issues have been discussed by other authors [11,15].

### 4.9. Oncologists Asked to Receive Their Patients’ Data in Aggregates

Oncologists’ suggestions to make the platform more user-friendly, to receive patients’ data in aggregates, as well as receive a message when a patient uploads a new file or an alert sign, have been taken into consideration. Efforts have been made to endorse patients’ and physicians’ suggestions in the 2nd step study within the SymbIASIS program “connecting hospitals with startups”.

### 4.10. Strengths

The strengths of the study were the successful patients’ registration and self-recording of their data, as well as the positive comments of patients and oncologists on the impact of the digital innovation on cancer care. This study “opened the door” to the next step, within the SymbIASIS program, which assesses patients in the hospital while on intravenous cancer therapy under the supervision of a dedicated study nurse.

### 4.11. Weaknesses

The small number of patients who were “selected” to participate in the study based on their self-reported internet literacy represents a weakness. The small number of patients may be related to the very new digital innovation model for cancer patients’ approach in 2020. Patients’ selection according to internet literacy might, at that time, be necessary for patients to use digital innovations.

Another weakness was the lack of recording the number of patients who were invited vs. those who accepted and participated in the study. The lack of financial support to hire a dedicated nurse to record patients’ participation was the reason for that weakness.

## 5. Conclusions

This early-stage feasibility study suggests that the CureCancer platform is feasible. It also revealed the need for educational programs to help older people and people of lower education use digital innovation.

## Figures and Tables

**Table 1:** Patient, disease, and treatment characteristics, *n* = 114.

Parameter		N	%
Gender	Male/Female	63/51	55.3/44.7
Age	Range (n)/Median	23–87/54.5	
Educational status			
	University	64	56.1
	High School	35	30.7
	Junior and Elementary Schools	15	13.2
Cancer type			
	Breast	23	20.2
	Lung	16	14.0
	Head/Neck/Oral	12	10.5
	Colorectal	10	8.8
	Pancreatic	9	7.9
	Other cancers *	21	18.4
	Not identified types	23	20.2
	Multiple cancers not specified	5	4.4
Patients with metastatic disease		46	40.4
Patients on cancer therapy		87	76.3
	Chemotherapy	48	42.1
	Immunotherapy	9	7.9
	Targeted therapy	4	3.5
	Radiotherapy	4	3.5
	Combined therapies	13	11.4
	Hormonal treatment	9	7.9
Supportive Care **			
	Any supportive care	51	44.7
	Antiemetics	22	19.3
	Pain medications	16	14.0
	Psychological support	15	13.2
	Bone targeting agents	13	11.4
	Medical diet	10	8.8
Patients visiting non-oncology HCPs			
	At least 1 HCP	114	100
	More than 1 HCP (2–9)	47	41.2
Total number of non-oncology HCPs visited by patients **		208	
Mean number of HCPs visited per patient		1.82	
Mean number of HCPs visited by patients on active treatment vs. no current treatment		1.92 vs. 1.51 (*p* = 0.18, *t*-test)	
Specialty of HCP			
	Cardiologist	44	38.6
	Dentist/Stomatologist	40	35.0
	Dietician	19	16.7
	Psychologist/Neurologist	18	15.7
	Endocrinologist	11	9.6
Patients with prior cancer therapies		81	71.0

* Sarcomas (*n* = 4), Hepatic (*n* = 3), Gastric (*n* = 3), Leukemia (*n* = 2), M. Myeloma (*n* = 2), Skin (*n* = 2). ** categories are not mutually exclusive.

**Table 2:** Patients experiences and responses, *n* = 114.

Patients Experiences and Responses, *n* = 114		
	No/A little	Much/Very much
	N (%)	N (%)
1 Was your registration and CureCancer use easy?	16 (14.0)	98 (86.0)
2 Was it easy to upload your files?	67 (58.8)	47 (41.2)
	No	Yes
	N (%)	N (%)
3 Do you prefer the digital way?	24 (21.1)	90 (78.9)
4 Will you recommend CureCancer use to others?	15 (13.2)	99 (86.8)

## Data Availability

All data generated or analyzed during this study are available to the Journal and the reviewers, as well as to any interested reader who asks.

## References

[1] (2020). The Cancer Atlas. https://canceratlas.cancer.org/the-burden/.

[2] American Cancer Society Cancer Action Network (2020). The Costs of Cancer. https://www.fightcancer.org/policy-resources/costs-cancer.

[3] Canadian Cancer Society. https://cancer.ca/en/research/cancer-statistics.

[4] Cancer Research UK. https://www.cancerresearchuk.org/health-professional/cancer-statistics/risk.

[5] Brandt J., Scotté F., Jordan K. (2019). Patient-reported outcomes (PROs) as a routine measure for cancer inpatients: The final missing piece of the puzzle?. Ann. Oncol..

[6] Mierzynska J., Piccinin C., Pe M., Martinelli F., Gotay C., Coens C., Mauer M., Eggermont A., Goenevold, Bjordal K. (2019). Prognostic value of patient-reported outcomes from international randomized clinical trials on cancer: A systematic review. Lancet Oncol..

[7] Bakouny Z., Hawley J.E., Choueiri T.K., Peters S., Rini B.I., Warner J.L., Painter C.A. (2020). COVID-19 and Cancer: Current challenges and perspectives. Cancer Cell.

[8] Burbury K., Wong Z.W., Yip D., Thomas H., Brooks P., Gilham L., Piper A., Solo I., Underhill C. (2021). Telehealth in cancer care: During and beyond the COVID-19 pandemic. Intern. Med. J..

[9] Basch E., Stover A.M., Schrag D., Chung A., Jansen J., Henson S., Carr P., Ginos B., Deal A., Spears B.S. (2020). Clinical utility, and user perceptions of a digital system for electronic patient-reported symptom monitoring during routine cancer care: Findings from the PRO-TECH Trial. JCO Clin. Cancer Inform..

[10] Bhargava R., Keating B., Isenberg S.R., Saranjan S., Chasen M. (2021). RELIEF: A digital health tool for the remote self-reporting of symptoms in patients with cancer to address palliative care needs and minimize emergency department visits. Curr. Oncol..

[11] Aapro M., Bossi P., Dasari A., Fallowfield L., Gascon P., Geller M., Jordan K., Kim J., Martin K., Porzig S. (2020). Digital health for optimal supportive care in oncology: Benefits, limits, and future perspectives. Support. Care Cancer.

[12] Guo C., Ashrafian H., Ghafur S., Fontana G., Gardner C., Prime M. (2020). Challenges for the evaluation of digital health solutions-A call for innovative evidence generation approaches. npj Digit. Med..

[13] Walker D.M., Hefner J.L., Fareed N., Huerta T., McAlearney A.S. (2019). Exploring the digital divide: Age and race disparities in use of an inpatient portal. Telemed. E-Health.

[14] Leader A.E., Capparella L.M., Waldman L.B., Cammy R.B., Petok A.R., Dean R., Shimada A., Yocavitch L., Rising K.L., Garber G.D. (2021). Digital literacy at an urban cancer center: Implications for technology use and vulnerable patients. JCO Clin. Cancer Inform..

[15] Dixit N., Van Sebille Y., Crawford G.B., Ginex P.K., Ortega P.F., Chan R.J. (2022). Disparities in telehealth use: How should the supportive care community respond?. Support. Care Cancer.

[16] Silberman J., Wicks P., Patel S., Sarlati S., Park S., Korolev I.O., Carl J.R., Owusu J.T., Mishra V., Kaur M. (2023). Rigorous and rapid evidence assessment in digital health with the evidence DEFINED framework. npj Digit. Med..

[17] Bottaro R., Faraci P. (2022). The influence of socio-demographics and clinical characteristics on coping strategies in cancer patients: A systematic review. Support. Care Cancer.

[18] StataCorp (2017). Stata Statistical Software, Release 15.

[19] Cuschieri S. (2019). The STROBE guidelines. Saudi J. Anaesth..

[20] Aljabri D., Dumitrascu A., Burton M.C., White L., Khan M., Xirasagar S., Horner R., Naessens J. (2018). Patient portal adoption and use by hospitalized cancer patients: A retrospective study of its impact on adverse events, utilization, and patient satisfaction. BMC Med. Inform. Decis. Mak..

[21] Hassan A.M., Chu C.K., Liu J., Angove R., Rocque G., Gallagher K.D., Momoh A.O., Caston N.E., Williams C.E., Wheeler C.E. (2022). Determinants of telemedicine adoption among financially distressed patients with cancer during the COVID-19 pandemic: Insights from a nationwide study. Support. Care Cancer.

[22] Sharma P., Sinicrope A.R., Sinicrope P., Brockman T.A., Reinicke N.M., West I., Wiepert L.M., Glasgow A.E., Sangaralingham L.R., Holland A.L. (2022). Patient Telemedicine perceptions during the COVID-19 pandemic within a multi-state medical Institution: Qualitative study. JMIR Form. Res..

[23] Tran C., Dicker A., Leiby B., Gressen E., Williams N., Jim H. (2020). Utilizing Digital Health to Collect Electronic Patient-Reported Outcomes in Prostate Cancer: Single-Arm Pilot Trial. J. Med. Internet Res..

[24] Potdar R., Thomas A., DiMeglio M., Mohiuddin K., Djibo D.A., Laudanski K., Dourado C.M., Leighton J.C., Ford J.G. (2020). Access to internet, smartphone usage, and acceptability of mobile health technology among cancer patients. Support. Care Cancer.

[25] Smith B., Magnani J.W. (2019). New technologies, new disparities: The intersection of electronic health and digital health literacy. Int. J. Cardiol..

[26] Patt D.A., Wilfong L., Toth S., Broussard S., Kanipe K., Hammonds J., Allen V., Mautner B., Campbell N., Dubey A.K. (2021). Telemedicine in community cancer care: How technology helps patients with cancer navigate a pandemic. JCO Oncol. Pract..

[27] Knudsen K.E., Willman C., Winn R. (2021). Optimizing the use of Telemedicine in Oncology care: Post pandemic opportunities. Clin. Cancer Res..

[28] Keats M.R., Cui Y., DeClercq V., Grandy S.A., Sweeney E., Dummer T.J.B. (2021). Burden of multimorbidity and polypharmacy among cancer survivors: A population-based nested case-control study. Support. Care Cancer.

[29] Suh Y., Ah Y.M., Lee E., Lee J.Y. (2021). Association of inappropriate polypharmacy with emergency department visits in older patients receiving anti-neoplastic therapy: A population-based study. Support. Care Cancer.

[30] Fraser A., McNeill R., Robinson J. (2022). Cancer care in a time of COVID: Lung cancer patient’s experience of telehealth and connectedness. Support. Care Cancer.

[31] Brady G., Ashforth K., Cowan-Dickie S., Dewhurst S., Harris N., Monteiro A., Sandsung C., Roe J. (2022). An evaluation of the provision of oncology rehabilitation services via telemedicine using a participatory design approach. Support. Care Cancer.

[32] Mols F., Tomalin B., Pearce A., Kaambwa B., Koczwara B. (2020). Financial toxicity and employment status in cancer survivors. A systematic literature review. Support. Care Cancer.

[33] Olver I., Keefe D., Herrstedt J., Warr D., Roila F., Ripamonti C. (2020). Supportive care in cancer—A MASCC perspective. Support. Care Cancer.

[34] Choi Y.J., Lee W.-Y. (2023). Unemployment risk of all employed working-age cancer survivors after cancer diagnosis in South Korea: A retrospective cohort analysis of population-based administrative data. Support. Care Cancer.

[35] Tremblay D., Touati N., Usher S., Gentil B., Courval M.-J. (2023). The challenge of optimizing supports for people living with and beyond cancer: Creating proximity between cancer and non-profit community-based providers. Support. Care Cancer.

[36] Neeman E., Kumar D., Lyon L., Kolevska T., Reed M., Sundaresan T., Arora A., Li Y., Seaward S., Kuehner G. (2021). Attitudes and perceptions of multidisciplinary cancer care clinicians toward telehealth and secure messages. JAMA Netw. Open.

[37] Turner K., Babilonia M.B., Naso C., Nquyen O., Gonzalez B.D., Oswald L.B., Robinson E., Lafata J.E., Ferguson R.J., Tabriz A.A. (2022). Health care providers’ and professional experiences with Telehealth Oncology implementation during the COVID-19 pandemic: A qualitative study. JMIR.

[38] Curran V.R., Hollett A., Peddle E. (2023). Virtual care and COVID-19: A survey study of adoption, satisfaction, and continuing education preferences of healthcare providers in Newfoundland and Labrador, Canada. Front. Digit. Health.

